# Functional evaluation of patients with surgically treated terrible triad of the elbow

**DOI:** 10.1590/1413-78522015230301008

**Published:** 2015

**Authors:** Rafael Mulatti Brigato, Guilherme Grisi Mouraria, Fernando Kenji Kikuta, Sérgio de Paula Coelho, Márcio Alves Cruz, Américo Zoppi

**Affiliations:** 1 Department of Orthopedics and Traumatology, Universidade Estadual de Campinas (Unicamp), Campinas, SP, Brazil

**Keywords:** Ulna fractures, Radius fractures, Dislocations, Elbow

## Abstract

**OBJECTIVES:**

To evaluate the functional outcome of patients with surgically treated terrible triad of the elbow.

**METHODS:**

A retrospective evaluation was performed using the MEPS score (Mayo Elbow Performance Score) of patients diagnosed with terrible triad of the elbow who underwent surgical treatment.

**RESULTS:**

14 patients (nine men and five women) and 15 elbows (one bilateral case) were evaluated. A MEPS average score of 78 points and 86% good and excellent results was obtained. As complications, we had one case of infection and three of neuropraxia of the ulnar nerve.

**CONCLUSION:**

The patients had stable elbow with good function, however with reduced range of motion. *Level of Evidence IV, Case Series.*

## INTRODUCTION

The terrible triad of the elbow (TTE) is the name given to elbow dislocation associated to fractures of the coronoid process of the ulna and the radial head. Besides bone injuries, the elbow dislocation may be associated with ligament injuries, specifically lateral collateral ligament, medial collateral ligament and anterior capsule, important articular stabilizers.^1^ These injuries lead to a high articular instability.^2^

The TTE may be caused by high and low energy trauma.^3^ The most common mechanism is the posterior dislocation of the elbow. It occurs from falls on the hand or wrist in supination and with the elbow in hyperextension associated with valgus stress. A resulting anterior strength leverages the ulna out of the humeral trochlea. The fracture of the coronoid process is a consequence from its impact against the trochlea. The fracture of the radial head is caused by the valgus stress to which the elbow has been submitted.^4^ This position also promotes failure of the ulnar lateral collateral ligament and consequent posterolateral dislocation of the radial head.^3^ In some cases, TTE caused by high kinetic energy trauma can evolve to rupture of the medial ligament of the elbow complex. The etiopathogenesis of these injuries was described by O'Driscoll.^5^

Conservative treatment has unsatisfactory outcomes, with joint stiffness, recurrent instability and joint osteoarthritis due to imobilization.^2^ The treatment of choice is surgery. The osteosynthesis of fractures and repair of ligament injuries allow the stabilization of the elbow joint and early mobility.^6^^,^ 7

The radial head fractures are preferably treated with osteosynthesis. When reconstruction of the fracture is not possible, usually when there is great comminution of the fragments, we performed resection of bone fragments and the radial head is replaced by a prosthesis.^1^

The coronoid fractures should be repaired. Type 1 injuries described by Morrey and O'Driscoll^8^ are usually treated with anterior capsular repair with transbone insertion in the ulna. In types 2 and 3 of Morrey and O'Driscoll^8^ classification osteosynthesis with support plates or interfragmentary screws are enough to stabilize the coronoid and hence the elbow, preventing posterior instability of the elbow.

The repair of ligament injuries is critical to maintaining the stability of the elbow. The repair of the lateral ligament complex is always needed, according to Hori cited by O'Driscoll et al.,^9 ^this structure is always the first to be damaged evolving with rotatory posterolateral instability. The medial ligament complex is explored and evaluated during surgery and when injured, it must be repaired.^5^^,^ 9 ^,^ 10

Surgical repair of bone and ligament injuries may evolve with decreased joint mobility, pain and eventually with instability, even when properly performed.^1^

The objective of this study was to evaluate the functional outcomes of patients with terrible triad of the elbow treated surgically. 

## MATERIALS AND METHODS

**Figure 1.**
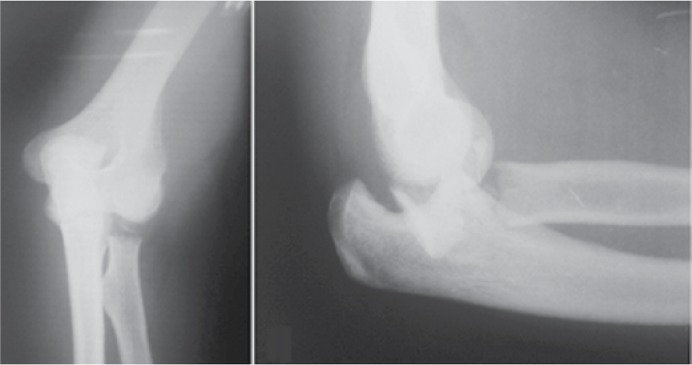
Profile and AP X-Ray showing the terrible triad of the elbow.

**Figure 2.**
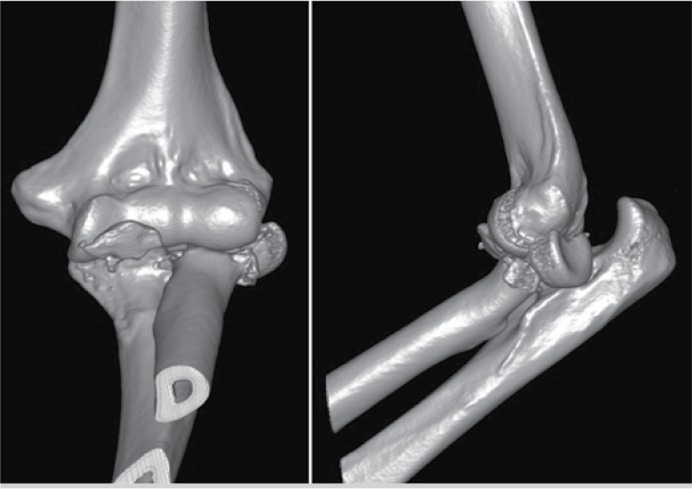
Computed Tomography (3D reconstruction) of the injury.

Fourteen patients with injuries in 15 elbows (1 bilateral case) diagnosed with terrible triad of the elbow were evaluated retrospectively between 2009 and 2013. In order to evaluate the clinical data, we used medical records and classification and to classify fractures, imaging exams such as radiography and computed tomography. (Figures 1 and 2)

Injury of the coronoid process was classified with the aid of computed tomography. We adopted the classification proposed by Morrey and O'Driscoll:^8^ Type I (impairment of the apex of the coronoid process), type II (commitment to 50% of the size of the coronoid process) and type III (when the injury is over 50% of the size the coronoid process).

In surgical repair, we used two-way access. We started the procedure always by the side track (Kocher) for osteosynthesis or the radial head arthroplasty and repair of the lateral ligament complex. The ligament repair was performed using 4 mm metal anchors placed in the central region of the lateral epicondyle of the humerus, at the isometric point, and points tensing the lateral capsule avoiding the appearance of rotatory posterolateral instability.

After the lateral repair, the anteromedial access to the elbow was performed. After isolation of the ulnar nerve by blunt dissection of the medial elbow muscle group and protecting the neurovascular bundle, the approach to the coronoid process was made. In fractures classified as Morrey types 1 or 2, we used 2.5 mm metal anchors, with transbone insertion of the anterior capsule of the elbow.

In fractures classified as type 3, osteosynthesis was performed with a 2.5mm molded board. If a medial ligament complex injury was detected, repair with 4 mm metal anchors was made, placed on the isometric point of the humeral lateral epicondyle.

In the postoperative period, the elbow was immobilized with axillary-palmar splint at ninety degrees flexion and medium prono-supination for two weeks. After this period, immobilization and stitches were removed and the patient was referred to physical therapy.

We evaluated the bone structures by radiological examination, observing the joint congruence, fracture healing, and the presence of intra or periarticular calcification or other change detectable by this image method. The range of motion and function of the elbow were also evaluated through the Mayo Elbow Performance Score (MEPS) scale. This scale assesses pain parameters, range of motion, stability and elbow function. Patients with scores greater than 90 are rated as excellent; those with scores between 75 and 89 are classified as good; between 60 to 74 they are classified moderate and patients with lower scores 60 points were classified as poor outcome.

The study was approved by the local Ethics Committee with approval number 27730414.0.0000.5404, consentment number 635.804.

All patients agreed to participate in the study through a Free and Informed Consent form. 

## RESULTS

We evaluated retrospectively the medical records of 14 patients and 15 elbows (one bilateral case).

The mean follow-up of patients was 14.8 months.

In 13 patients with radial head fracture with great bone comminution, osteosynthesis was not possible, and we decided to stabilize the elbow with a prosthesis. In two cases the osteosynthesis was feasible, and the fragments were stabilized with 2.5 mm screws.

Damage to the lateral ligament complex occurred in all patients. They were repaired with the aid of an anchor. The repair of the medial collateral ligament was performed in 11 of the 15 elbows (73% of cases).

The 13 elbows with fracture of the coronoid process classified as Morrey type I (85% of cases) were subjected to capsule reconstruction using anchors. In two elbows fractures were classified as type II, and bone fragments were fixed with plate and screws.

**Figure 3.**
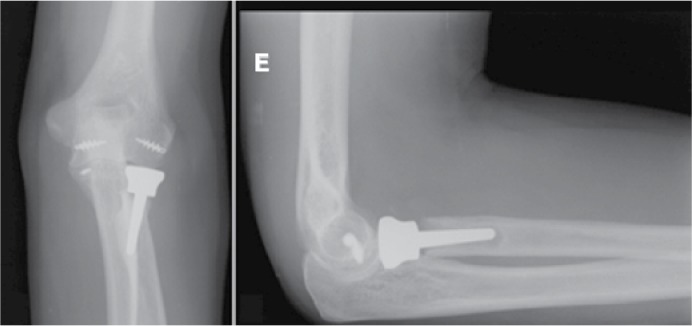
Postoperative incidence X-Ray.

The treatment adopted in most cases was the replacement of the radial head by a prosthesis associated with repair of the coronoid process and reconstruction of the lateral ligament and medial elbow complex with the aid of anchors. (Figure 3)

The arc of movement of the elbow observed after the end of treatment was 98.66° ± 25.87° in flexion-extension and 80.33° ± 30.26° in pronation-supination. In no elbow we observed clinical signs of joint instability. (Table 1)

**Table 1.** Age and arc of movement postoperatively.

Patient	Age (years old)	Time (months)	Flexion	Extension	Pronation	Supination
1	51	15	160	0	80	60
2	44	9	120	20	30	40
3	39	14	100	20	60	30
4	48	60	100	10	60	20
5	29	10	110	10	70	30
6	34	12	100	10	70	35
7	30	36	100	20	10	20
8	42	10	120	10	45	50
9	42	10	110	20	30	20
10	38	6	120	10	45	30
11	57	15	110	10	30	40
12	31	6	110	20	40	45
13	29	4	130	10	50	45
14	52	3	90	50	10	10
15	19	12	130	10	70	30
Mean / St. Dev.	39 ± 10.46	14.8 ± 14.69	114 ± 17.23	15.33 ± 11.25	46.66 ± 21.84	33.66 ± 13.29

**Figure 4.**
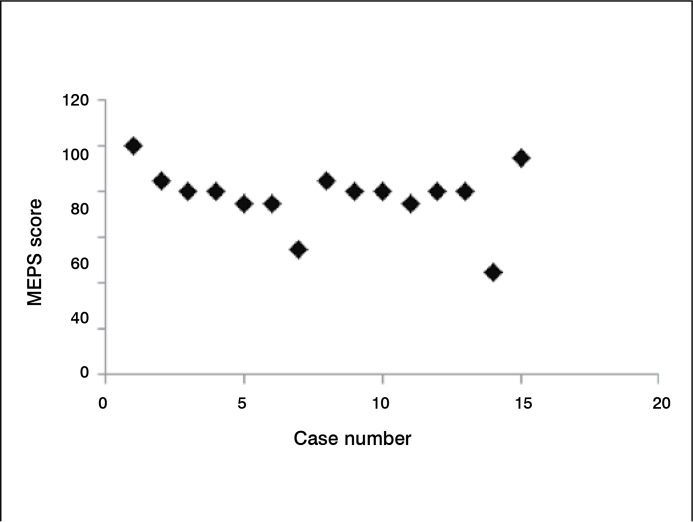
MEPS Score per patient.

In two elbows the final outcome evaluated by MEPS scale was considered excellent. According to this criterion, in 10 elbows results were classified as good. Poor outcome was observed in only two elbows treated in this series. (Table 2 and Figure 4)

**Table 2.** Postoperative MEPS Scores.

Patient	Pain	Arc of Movement	Stability	Function	Total MEPS
1.	45	20	10	25	100
2.	30	20	10	25	85
3.	30	15	10	25	80
4.	30	15	10	25	80
5.	30	15	10	20	75
6.	30	15	10	20	75
7.	15	15	10	15	55
8.	30	20	10	25	85
9.	30	15	10	25	80
10.	30	15	10	25	80
11.	30	15	10	20	75
12.	30	15	10	25	80
13.	30	15	10	25	80
14.	15	5	10	15	45
15.	45	20	10	20	95
Mean / St. Dev.	30 ± 8.01	15.66 ± 3.71	10 ± 0	22.33 ± 3.71	78 ± 13.46

As complications, we had one case of superficial infection, that has been treated with antibiotics. Three patients had ulnar nerve neuropraxia presenting full recovery within four months. 

## DISCUSSION

The terrible triad of the elbow is a serious and potentially disabling injury. It is caused by high kinetic energy trauma, usually in young patients.^10^ In this series we observed that males were the majority, representing 64% of patients. The mean age was 38 years and 8 months old. These data are similar to other authors'.^1^

The fractures of the coronoid process classified according to Regan & Morrey as type I represented the majority (85%) of cases, the other 15% were type II and there were no type III cases. Our series is similar to that described by a work from 2011^2^ and others from 2005^8^ and 2014.^12^

The goal of surgical treatment of TTE is to stabilize the elbow. We must perform bone and ligament reconstruction allowing joint mobility as early as possible. The bone stabilization is achieved with the reconstruction of the coronoid process.^7^ In this series, in all cases we performed the reconstruction of the coronoid process independently of the type of fracture.

The radial head is a secondary stabilizer of stress in elbow valgus. Its preservation is recommended whenever possible, however, when its reconstruction is not possible, then its prosthetic replacement is adviced.^12^ In this patients series, this was done in 86% of the elbows. In two patients osteosynthesis was feasible. This can be explained by a strong fragmentation of the radial head, preventing a stable fixation.

Injuries of the lateral collateral ligament were observed in all patients. This ligament complex is the first to be compromised in the elbow dislocation as described by Shawn et al.^13^ In our series all patients underwent surgical repair of the lateral elbow stabilizers, as recommended in the literature.^1^

The injury of the medial collateral ligament (MCL) was found in 10 cases (71%). These patients underwent surgical repair. According to some authors, the MCL repair should be performed in cases of residual instability. And even after the repair of the MCL, if the articulation remains unstable, placement of a dynamic trans-acetabular external fixator should be performed.^1^^,^ 7

In patients treated, after the repair of the MCL, the elbow remained stable with no need of using external fixation.

In this series, with the treatment adopted for our patients, we obtained good final outcome (86%). All elbows were stable. Pain at rest or in motion was not reported by the patients. The main complication was partial mobility loss observed mainly during prono-supination. Despite this limitation, all treated elbows had a functional arc of movement, allowing the performance of activity of daily living. Our results are comparable to the literature's.^2^^,^ 7

It is essential that the patient start physical therapy immediately, especially active and passive kinesiotherapy aiming to gain articular range of motion. In the patients treated, many times access to that kind of treatment was impossible due to socioeconomic status (difficulty in attending a physiotherapy clinic, losing workdays and others).

Regarding complications, we had one case of superficial infection (6%) and three cases of neuropraxia of the ulnar nerve (20%). All of them had a satisfactory outcome. For comparison, Rodriguez-Martin et al.^1^ reported infection rates between 2 and 5% and ulnar nerve dysfunction between 10 and 22%.^1^

## CONCLUSION

Patients undergoing surgical treatment of terrible triad of the elbow evolved with stable elbow, with good function, but with decreased range of motion.
